# Dynamic microscale flow patterning using electrical modulation of zeta potential

**DOI:** 10.1073/pnas.1821269116

**Published:** 2019-05-06

**Authors:** Federico Paratore, Vesna Bacheva, Govind V. Kaigala, Moran Bercovici

**Affiliations:** ^a^IBM Research–Zurich, 8803 Rüschlikon, Switzerland;; ^b^Faculty of Mechanical Engineering, Technion–Israel Institute of Technology, 3200003 Haifa, Israel;; ^c^Department of Mechanical Engineering, The University of Texas at Austin, Austin, TX 78712

**Keywords:** electrokinetics, microfluidics, electroosmotic flow, viscous flow, Hele–Shaw cell

## Abstract

The ability to move fluids at the microscale is at the core of many scientific and technological advancements. Despite its importance, microscale flow control remains highly limited by the use of discrete channels and mechanical valves, and relies on fixed geometries. Here we present an alternative mechanism that leverages localized field-effect electroosmosis to create dynamic flow patterns, allowing fluid manipulation without the use of physical walls. We control a set of gate electrodes embedded in the floor of a fluidic chamber using an ac voltage in sync with an external electric field, creating nonuniform electroosmotic flow distributions. These give rise to a pressure field that drives the flow throughout the chamber. We demonstrate a range of unique flow patterns that can be achieved, including regions of recirculating flow surrounded by quiescent fluid and volumes of complete stagnation within a moving fluid. We also demonstrate the interaction of multiple gate electrodes with an externally generated flow field, allowing spatial modulation of streamlines in real time. Furthermore, we provide a characterization of the system in terms of time response and dielectric breakdown, as well as engineering guidelines for its robust design and operation. We believe that the ability to create tailored microscale flow using solid-state actuation will open the door to entirely new on-chip functionalities.

## Significance

Traditional microfluidic devices make use of physical channels and mechanical actuators, in which geometries and functionalities are intimately related to one another, i.e., changing the flow field requires change at the mechanical level. In this work, we introduce a concept in which a microfluidic chamber with no preset structures or active mechanical components can be dynamically configured to produce desired flow fields.

Manipulation of fluids at the microscale is important for a wide range of applications, from laboratory-on-a-chip devices (1), through adaptive optics (2, 3), to energy harvesting (4, 5). Whereas two-phase flows can be controlled by acting on a fluid–fluid interface through a variety of physical mechanisms [e.g., electrowetting on dielectric (6), dielectrophoresis (7), and thermocapillary (8)], control of continuous-phase flows at the microscale, lacking such an interface, remains a significant challenge.

At present, routing single-phase flows through desired flow paths is obtained primarily by the use of solid channels, constraining the types of flows that can be achieved. The development of on-chip valves (9) increased the flexibility of routing fluids on a large scale, allowing complex and dynamic fluid manipulation through microfluidic networks. However, valve-based platforms still rely on fixed-channel geometries, are limited to discrete actuation, and are not easily scalable.

Electroosmotic flow (EOF) is the motion of an electrolyte resulting from the interaction of an external electric field with the net charge of an electric double layer (EDL). Recently, Boyko et al. (10) suggested theoretically that nonuniform EOF can be used to achieve desired flow patterns in planar configurations. Paratore et al. (11) experimentally demonstrated this concept using chemical patterning of the surface by deposition of polyelectrolytes. Because the zeta-potential pattern is obtained by chemical modification, this approach can only generate static flow fields. Moreover, the number of flow patterns that can be created is severely limited by the discrete number of zeta potential that can be prescribed (i.e., number of available polyelectrolytes).

An alternative approach to control the EOF relies on the application of a perpendicular electric field to the surface, thus enabling modification of the zeta potential in a dynamic fashion. The use of an external dc field for modifying zeta potential was first proposed by Ghowsi and Gale (12) and demonstrated experimentally by Lee et al. (13) in the context of capillary electrophoresis to reduce band broadening and adsorption of analytes on capillary walls (14–16). Schasfoort et al. (17) were the first to implement this approach on-chip and propose it as a controlling and switching element in microfluidic networks, followed by several works exploring the effect of different dielectric layers (17–21). Several names were proposed for this phenomenon including “flow field-effect transistor (flowFET)” (17) and “field-effect flow control” (18); herein we will refer to it as “field-effect electroosmosis” or FEEO as it best captures the physical phenomenon, as was originally proposed in 1989 by Ghowsi and Gale (12).

FEEO is most effective when working at pH values close to the point of zero charge of the substrate, at which the native zeta potential of the surface is approximately zero, and the field effect becomes predominant. At higher or lower pHs, FEEO has a smaller contribution relative to the native surface potential (22). Moreover, because the process is driven by a dc electric field, gas bubble due to electrolysis can limit its use, and the fluid flow cannot be used to directly dictate the motion of molecules and particles in the liquid as those also experience electrophoretic migration. While for native surfaces an ac driving field would lead to a zero time-averaged EOF, Muthu et al. (23) showed that by synchronizing the phase of a gate electrode with that of the driving field, a net flow in a desired direction can be obtained. More recently, van der Wouden et al. (24, 25) investigated the time response of such systems and demonstrated its application to microfluidic pumping.

Here we report the use of ac-FEEO as a mechanism for dynamic control of spatial flow patterns in microfluidic chambers. Using a discrete disk-shaped gate electrode, we first reproduce the EOF dipole predicted by Boyko et al. (10). In contrast to chemical patterning (11), which has a fixed zeta-potential distribution, we show that the use of ac-FEEO allows a continuous range of zeta-potential values to be prescribed, thus enabling the flow field to be tuned in real time. We characterize the dielectric breakdown threshold for a range of dielectric materials and investigate the time response of our devices, providing engineering guidelines for the design of such systems. We then demonstrate the use of various electrode configurations for dynamically shaping microscale flows, creating dipoles, quadrupoles, and isolated flow regions, as well as the deformation of pressure-generated streamlines.

## Concept of Flow Patterning Using ac-FEEO

**Fig. 1.**
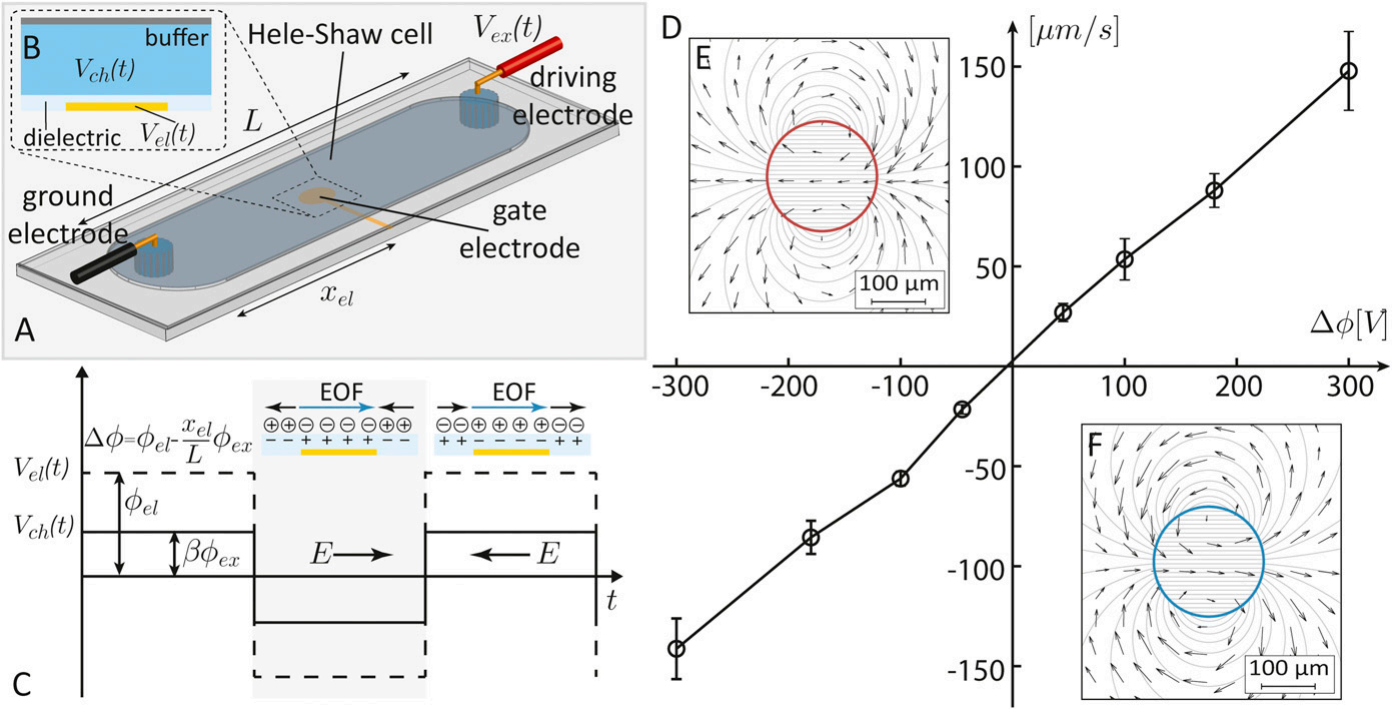
Concept of shaping EOFs by using gate electrodes in a Hele–Shaw cell. (*A*) Illustration of the fluidic system consisting of a Hele–Shaw cell of length L, a ground- (*Left*) and a driving (*Right*) electrode placed in two separate reservoirs, and gate electrodes placed at a distance $x_{el}$ from the ground electrode. (*B*) The gate electrode consists of a conductive layer insulated from the electrolyte in the chamber by a thin dielectric layer. (*C*) The potential of the driving electrode is set to a symmetric square-wave form of amplitude $\phi_{ex}$. The gate-electrode potential is modulated in sync with the driving potential with an amplitude $\phi_{el}$, resulting in a net EOF with intensity and directionality depending on the local amplitude difference $\Delta \phi =\phi_{el}-(x_{el}/L)\phi_{ex}$. The wave forms are here illustrated for the case of Δϕ>0, for which the EOF is directed toward the driving electrode. (*D*) Experimental results showing the depth-averaged velocity as function of the applied gate potential for an electric field amplitude of $\phi_{ex}/L=150V/\text{cm}$ as measured using a unidirectional flow configuration, as described in *Materials and Methods*. (*E* and *F*) Experimental (vector field) and analytical (streamlines) flow field of an EOF dipole generated by a 200-µm-diameter disc-shaped gate electrode for a negative and a positive Δϕ. Here the longest vector corresponds to a maximum velocity of 4.22 µm/s.

As illustrated in Fig. 1, we use a Hele–Shaw chamber of thickness *h* and length *L* filled with an electrolyte in direct contact with a ground electrode and a driving electrode (Fig. 1*A*). The floor of the chamber contains an embedded electrode (gate electrode) with a characteristic dimension $r_{0}$, located at a distance $x_{el}$ from the ground electrode. The gate electrode is electrically insulated from the electrolyte by a dielectric layer of thickness d and dielectric constant $\varepsilon_{d}$ (Fig. 1*B*). We actuate both the driving and the gate electrode with an ac potential at a frequency ω and amplitudes $\phi_{i}$, $V_{i}\left(t\right)=\phi_{i}f\left(\omega t\right)$, where the subscript i indicates either the external (ex) or the gate electrode (el) potential. Assuming a thin electric double-layer regime (26, 27), and assuming that 1/ω is longer than the charging time of the electric double layer, the EO slip velocity can be described by the Helmholtz–Smoluchowski relation (28),

$$u_{EOF}\left(t\right)=-\frac{\varepsilon_{l}\zeta \left(t\right)}{\eta }E\left(t\right),$$ [1]

where $\varepsilon_{l}$ is the dielectric permittivity of the liquid, η is its viscosity, $E\left(t\right)=V_{ex}\left(t\right)/L$ is the electric field in the chamber, and ζ(t) is the zeta potential relative to the bulk. For $r_{0}<<L$, this bulk potential can be assumed to be uniform over the electrode and given by $V_{ch}\left(t\right)=\left(x_{el}/L\right)\;V_{ex}\left(t\right)$. Regardless of the specific model used to describe the electric double layer [e.g., Guoy-Chapman, Stern, Bockris, etc. (ref. 28)], the zeta potential can be described by some function G of the difference between the gate and this bulk potential, $\zeta \left(t\right)=G\left(V_{el}\left(t\right)-V_{ch}\left(t\right)\right)$. Substituting the expression for E(t) and ζ(t) into Eq. **1**, the time-dependent EOF velocity can be expressed as

$$u_{EOF}\left(t\right)=-\frac{\varepsilon_{l}}{\eta L}\phi_{ex}f\left(\omega t\right)G\left[\left(\phi_{el}-\frac{x_{el}}{L}\phi_{ex}\right)f\left(\omega t\right)\right].$$ [2]

The time-averaged EOF velocity is obtaining by integrating Eq. **2** over one time period which, for the case where f is a square-wave function (Fig. 1*C*), yields

$$u_{EOF}^{av}\left(\Delta \phi \right)=-\frac{1}{2}\frac{\varepsilon_{l}}{\eta L}\phi_{ext}\left[G\left(\Delta \phi \right)-G\left(-\Delta \phi \right)\right],$$ [3]

where $\Delta \phi =\phi_{el}-\left(x_{el}/L\right)\phi_{ex}$ is the difference between the gate potential and bulk potential amplitudes. Eq. **3** shows that $u_{EOF}^{av}$ is a symmetric function of Δϕ regardless of the behavior of the function G. Fig. 1*D* shows an experimental measurement of the time-averaged EOF velocity as a function of the amplitude difference Δϕ, exhibiting the expected symmetry. We note that Eq. **3** is not valid outside the electrode region, where Δϕ is not defined. In such regions the zeta potential can be assumed to be constant in time and related only to the native surface charge, and the time-averaged EOF therefore vanishes. In *SI Appendix* we provide a more generalized formulation of the system, accounting also for dc biases.

To achieve flow patterning using ac-FEEO, we use one or more spatially distributed gate electrodes. In the lubrication approximation $\left(L>r_{0}\gg h\right)$, any discrete electrode (regardless of its specific shape) results in dipole-like circulation flow (*SI Appendix*, Fig. S6). Therefore, the choice of a disk-shaped electrode is a natural one as the depth-average velocity $\vec{u}$ can be described by a simple analytical expression (10),

$$\vec{u}\left(r,\theta ,\Delta \phi \right)=\left\{ \begin{array}{l} -\frac{\varepsilon_{l}}{4\eta L}\phi_{ext}\frac{\zeta \left(\Delta \phi \right)}{2}{\left(\frac{r_{0}}{r}\right)}^{2}\left[\cos\theta \hat{r}+\sin\theta \hat{\theta }\right]\;r>r_{0}\\ -\frac{\varepsilon_{l}}{4\eta L}\phi_{ext}\frac{\zeta \left(\Delta \phi \right)}{2}\left[\cos\theta \hat{r}-\sin\theta \hat{\theta }\right]\;r\leq r_{0}, \end{array}\right.$$ [4]

where r is the radial vector relative to the center of the disc and θ is the angle between r and the electric field. In our experimental setup h=15μm, $r_{0}∼100\mu \text{m}$, and L∼1 cm, satisfying the lubrication conditions.

Fig. 1 *E* and *F* shows the stream function (obtained from Eq. **4**) for a negative and a positive Δϕ, respectively, together with the experimentally measured vector flow field, for a 200-µm-diameter disc-shaped gate electrode. This illustrates that the expected dipole flow can be obtained using the ac-FEEO mechanism and that the intensity and direction of the dipole can be indeed tuned by controlling Δϕ. Movie S1 presents the visualization of this flow field during dynamic variation of Δϕ.

## System Design and Characterization

Central to the operation of the ac-FEEO is the ability to maintain capacitive charging over the gate electrode for a large number of charge and discharge cycles under high-driving electric fields (order of 100 V/cm). Clearly, a thick dielectric layer would be ideal to insulate the electrode against Faradaic currents, thus preventing bubble formation and pH changes resulting from electrolysis. However, the thickness of the dielectric layer should also be chosen to maximize the effect of the gate electrode on the induced zeta potential. A good approximation for the surface zeta potential as a function of Δϕ can be obtained from a capacitor model (12, 13, 15, 22, 28) accounting for the capacitance of the EDL $\left(C_{EDL}=\varepsilon_{l}A/\lambda_{EDL}\right)$ in series with the dielectric capacitance $\left(C_{d}=\varepsilon_{d}A/d\right)$,

$$\zeta =\zeta_{0}+\frac{C_{d}}{C_{EDL}}\Delta \phi =\zeta_{0}+\frac{\lambda_{EDL}}{\varepsilon_{l}}\varepsilon_{d}\frac{\Delta \phi }{d},$$ [5]

where $\zeta_{0}$ is the native zeta potential of the surface, and *λ*_*EDL*_ is the Debye length. Therefore, the most effective FEEO can be expected for a dielectric with the smallest possible thickness d and the largest possible permittivity $\varepsilon_{d}$. The best dielectrics can be obtained in standard microfabrication processes that (29) exhibit dielectric breakdown values on the order of 1 V/nm. Given that the EOF driving voltages in our system are in the range of 100–400 V, a 500-nm layer of a high-quality dielectric is expected to withstand such potential differences.

**Fig. 2.**
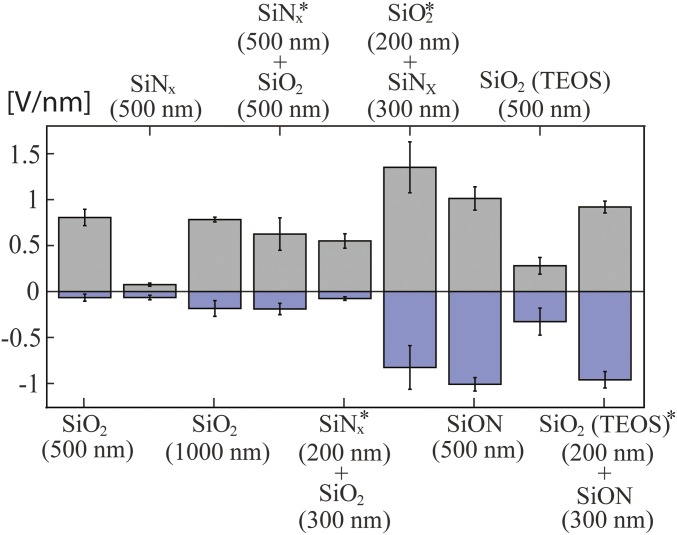
Experimental characterization of the breakdown strength of different dielectric layers deposited with PECVD. For the case of two materials, the material indicated with an asterisk forms the layer in direct contact with the metal. The SiN_*x*_ layer shows the worst performance with a breakdown of ∼0.1 V/nm; the SiO_2_ layer shows an asymmetric behavior, holding up to ∼0.8 V/nm for positive potential (Δϕ>0) but performing poorly for negative ones (Δϕ<0). Using a layer of SiO_2_ covered with a layer of SiN_*x*_ boosts significantly the breakdown strength, up to ∼1.3 V/nm for positive voltages; however, it shows poor repeatability (high error bars) and scarce symmetry. The best performance is obtained using SiON, withstanding up to ∼1 V/nm for both positive and negative voltages; therefore we choose SiON as insulating dielectric layer. The error bars represent the 95% confidence interval of the mean (with at least 10 repetitions).

Fig. 2 shows the measured dielectric breakdown field (breakdown voltage normalized by the dielectric thickness) for different dielectric coatings deposited with plasma-enhanced chemical vapor deposition (PECVD). See *SI Appendix*, Fig. S5 for details of the experimental setup and additional measurements for layer deposited with atomic layer deposition. The dielectric is inherently in an asymmetric configuration as it is in contact with a metal on one side and an electrolyte on the other. Because under an ac field the dielectric will be subjected to both positive and negative voltages, it is important to measure its breakdown for both cases. Pure SiN_*x*_ shows poor dielectric resistance, holding only up to ∼0.1 V/nm.

SiO_2_ performs significantly better for positive voltages, yet exhibits a clear asymmetry with a very low breakdown threshold for negative voltages (∼0.1 V/nm). Doubling the thickness of this layer to 1 µm does not show an improvement. A two-layer composition of SiN_x_ on top of SiO_2_ provides a significant improvement in both breakdown voltage and symmetry; however, we note that flipping the order of the layers is not equivalent and yields poor performance, even for large thicknesses. SiO_2_ deposition using tetraethyl orthosilicate precursors show better symmetry but a lower absolute breakdown field. The dielectric that showed the best performance is SiON, yielding a 1-V/nm breakdown voltage for both positive and negative applied voltages; therefore, we use this layer composition as the baseline of this work and cover it with an additional 100-nm layer of SiO_2_ because it has been well characterized for its EO properties (28).

The principle of ac-FEEO relies on the ability to synchronize the net charge in the double layer with the phase of the driving electric field. The upper bound on the frequency of the driving electric field is therefore dictated by the EDL charging time, which is a function of both the charge relaxation time within the electrolyte and any resistor–capacitor timescales associated with the electronics. Higher frequencies lead to reduced EOF as shown by van der Wouden et al. (24). *SI Appendix*, Fig. S5 presents the response of our system to a sudden change of applied gate voltage for different gate-electrode dimensions (see experimental details in *SI Appendix*). The observed timescale for the electrodes used in this work (200 × 200 µm), defined as the time needed for the gate current to drop to 10% of its initial value, is ∼5 ms. The maximum ac frequency that could be used while still benefiting from a fully charged EDL is ∼200 Hz, and we use this value as a frequency upper bound when operating our system. We note that the response of the system to positive and negative potentials is highly symmetric and we attribute this to the properties of the SiON layer used. This is in contrast to the asymmetric behavior of the SiO_2_ layer reported by van der Wouden et al. (25). A key advantage of using gate electrodes for controlling flows is the ability to switch from one flow pattern to another. The timescale associated with such switching is limited not only by the EDL charging process but also by the viscous response, $\tau_{v}=h^{2}/\nu$, where ν is the kinematic viscosity of the liquid. For a 15-µm-high chamber and an aqueous solution, as used in our experiments, $\tau_{v}∼0.2\;\text{ms}$, significantly shorter than the electric response, thus not limiting the switching time. However, for h ∼ 100 µm, $\tau_{v}$ is on the same order of magnitude of the EDL charging and could dominate the dynamic response of the system.

**Fig. 3.**
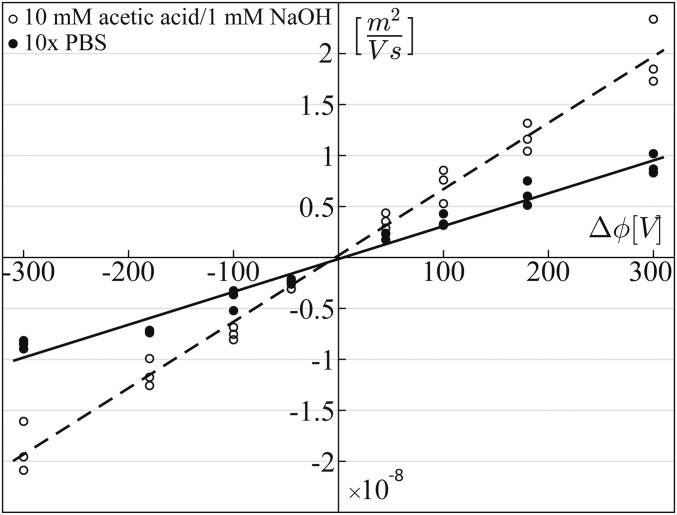
Experimental results showing the time-averaged EO wall mobility as function of Δϕ for different buffers: 10 mM acetic acid/1 mM NaOH (pH 3.8, 1.4 × 10^−2^ S/m, dashed line) and 10-times diluted PBS (pH 7.4, 2 ×10^−1^ S/m, continuous line). In this range of applied potentials, as expected from Eq. **5**, both curves show a linear dependence, with R^2^ values of 0.96 and 0.99, respectively. The experiments are performed using a straight channel with an array of gate electrodes and fluorescent beads to trace the flow; the EO wall mobility is derived by the depth-average velocity obtained by PIV.

To complete the characterization of our system, Fig. 3 presents the measured time-averaged EO wall mobility $\left(\mu_{EO}^{av}=u_{EOF}^{av}/E\right)$ as a function of the applied amplitude difference Δϕ. While, as expected, higher EO wall mobility is obtained using a low-pH buffer (10 mM acetic acid and 1 mM NaOH, pH 3.8), the use of physiological pH buffer (10 times diluted PBS) also provides significant EO mobility, indicating the potential use of ac-FEEO flow patterning for biochemical applications. Both curves show a linear dependence within this range of applied potentials, consistent with Eq. **5**.

## Dynamic Flow Patterning

**Fig. 4.**
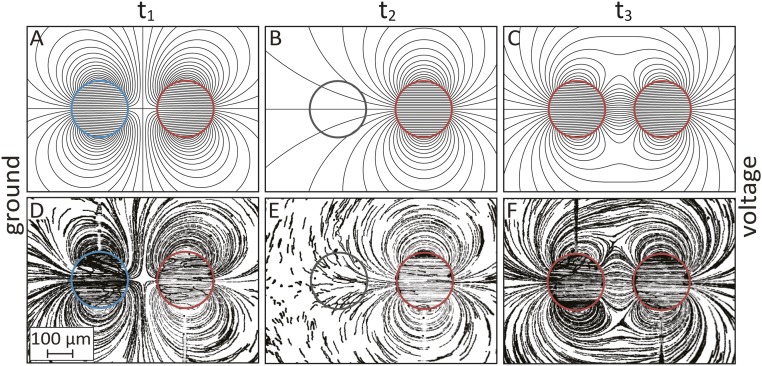
Analytical predictions and experimental visualization of flow streamlines generated by two 200-µm-diameter disc-shaped gate electrodes for different Δϕ combinations. (*A* and *D*) At t_1_, we set the electrodes to opposite Δϕ values, generating opposing dipoles and resulting in a quadruple flow field. (*B* and *E*) At t_2_, we switch the Δϕ of the left electrode to zero, effectively eliminating its effect on the flow. (*C* and *F*) At t_3_, we match the Δϕ of both electrodes, resulting in a flow configuration having two regions of local recirculation nested within a larger recirculating flow. The driving amplitude is $\phi_{ex}=200\;\text{V}$, and because the electrodes are located at the center of the chamber $\phi_{ch}∼100\;\text{V}$. A time-lapse movie showing the flow fields and the transition between them is provided in Movie S2.

Flow-field patterns can be obtained by superposition of flows generated by a distributed set of gate electrodes. The ability to individually address each electrode and dynamically modify its associated zeta potential allows switching from one flow pattern to another in real time. Fig. 4 demonstrates this concept for a basic case of two 200-µm-diameter disc-shaped gate electrodes. At t_1_ (Fig. 4*D*), the electrodes are assigned Δϕ values of +80 V (left electrode) and –80 V (right electrode), resulting in two dipoles with equal and opposite strengths, generating an EOF quadrupole. At t_2_ (Fig. 4*E*), we change the Δϕ value on the left electrode to 0 V, thus effectively eliminating its influence. At t_3_ (Fig. 4*F*), we match the Δϕ of the left electrode to the one on the right, resulting in a nested dipole configuration, consisting of recirculating flow around each electrode and a larger-scale recirculation between the two electrodes. The images presented here correspond to three time points of a continuous movie provided as Movie S2. These flow patterns can be well predicted by using Eq. **4** as shown in Fig. 4 *A*–*C*.

**Fig. 5.**
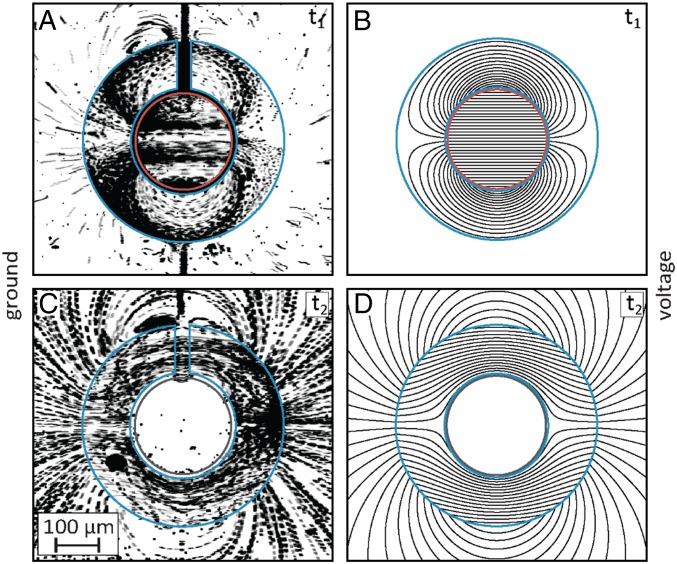
Analytical predictions and experimental visualization of flow streamlines generated by a 200-µm-diameter disc-shaped electrode surrounded by a 400-µm-outer-diameter annulus, for different Δϕ combinations. (*A* and *C*) At t_1_, the potential amplitude ratio of the two gate electrodes is set such that outside the annulus the two EOF dipoles flows cancel each other (inner Δϕ=−120 V and outer Δϕ=40 V), resulting in an isolated region of recirculating flow surrounded by a quiescent liquid. (*B* and *D*) At t_2_, we set the Δϕ of the disc electrode to zero, and demonstrate the opposite case of a finite stagnation volume surrounded by flow. The amplitude in the channel for both case is $\phi_{ex}=300\;\text{V}$, and approximately $\phi_{ch}=160\;\;\text{V}$ in the electrode region. A time-lapse movie showing the flow fields and the transition between them is provided in Movie S4.

A particularly interesting configuration consists of two concentric electrodes, i.e., a disc-shaped electrode (inner) of radius $R_{in}$ surrounded by an annulus-shaped one (outer) having an outer radius $R_{out}$. For such a case, theory predicts that setting $\Delta \phi_{in}=\left[{\left(R_{out}/R_{in}\right)}^{2}-1\right]\Delta \phi_{out}$ would result in an internal recirculation while maintaining zero velocity outside the outer electrode (Fig. 5*B*). Fig. 5*A* demonstrates the implementation of this configuration, showing that such a bounded flow field is indeed feasible. Some “leakage” of the flow field is however observed in the experiments; this is due to the imperfection of the annulus shape that contains a slit serving as a path for the electrical connection to the inner disc. Furthermore, the existence of the electrical lines themselves adds an additional perturbation to the flow. These perturbations also exist in Fig. 4 but they are less visible there because the velocity field magnitude is substantial compared with such perturbations. Setting $\Delta \phi_{in}=0\;\text{V}$ (Fig. 5 *C* and *D*) switches the flow field to a unique configuration in which a pressure jump at the outer edges of the annulus is compensated by an opposite pressure jump at the inner edges, leaving the inner region free of both slip velocity and pressure gradients. As a result, in the inner region the velocity field is uniformly zero, thus creating a finite stagnation volume within the flow.

## Dynamic Streamlines Shaping

**Fig. 6.**
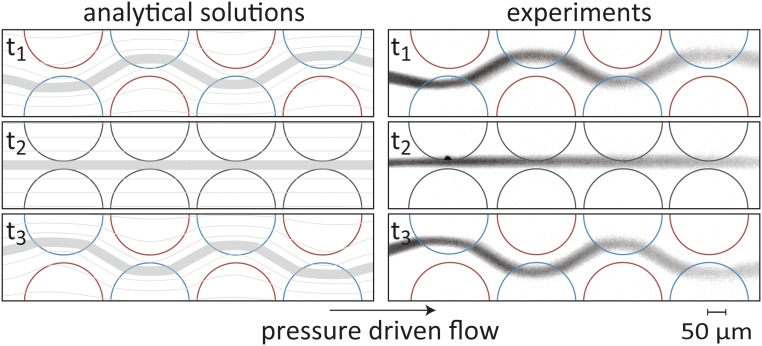
Analytical predictions and experimental visualization of flow-field shaping using a 2 × 4 array of gate electrodes. We establish a uniform flow in the chamber by pressure gradient and at t_1_, we set the electrode potentials to a checkerboard pattern with values of Δϕ=150 V and Δϕ=−150 V and an external amplitude to $\phi_{ex}=300\;\text{V}$. The multiple dipoles superpose with the uniform flow, resulting in sinusoidal shaping of the central streamline. When the gate potentials are set to zero (t_2_) the flow relaxes to its original shape. At t_3_, we flip the original checkerboard pattern, obtaining a sinusoidal shape with a shifted phase. The gate potential values used in the experiments are derived from theoretical predictions together with the calibration curve of Fig. 3, with no additional fitting parameters. A time-lapse movie showing the evolution of the streamline formation is provided in Movie S5.

A set of gate electrodes can also serve as an effective way of shaping existing flow fields created by, for example, pressure-driven flow or dc EOF. Fig. 6 shows the effect of a 2 × 4 array of disc-shaped electrodes on uniform flow generated by a pressure gradient. At t_1_, we activate the electrode array, assigning two values of Δϕ, a positive and a negative one, in a checkerboard pattern. As expected from our theoretical predictions, the velocity components perpendicular to the electric fields bend the incoming flow, resulting in a sinusoidal streamline. At t_2_, we set Δϕ=0 to all electrodes and the flow field relaxes back to its original state. At t_3_, we invert the checkerboard pattern, resulting again in a sinusoidal shape but with a phase shifted by 180°. In the simulation, the incoming velocity field was set to 40 µm/s to match the experiments, and the slip velocities of the electrodes were tuned to obtain the desired flow pattern. Through the use of the independent measurements of the EO wall mobility reported in Fig. 3, these slip velocities were translated to Δϕ values, which were then used in the experiments with no additional fitting parameters. As shown in Fig. 6, the agreement between theory and measurements is not only qualitative but also quantitative, showing the streamlines being deflected to the same extent.

**Fig. 7.**
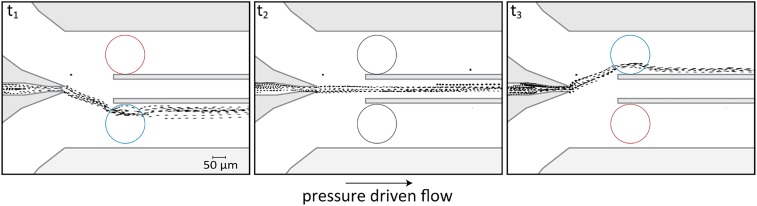
Demonstration of flow-field shaping integrated in a microfluidic device. The gate electrodes can be used to shape an existing flow field and direct an incoming streamline into one of the three outlets. At t_1_, we impose Δϕ=−310V and Δϕ=350 V to the top and bottom electrode, respectively, to induce a counterclockwise velocity field, thus pushing the streamline to the bottom outlet. At t_2_, we set a Δϕ=0 to both electrodes and the flow returns to its native state, with the central streamline continuing to the central outlet undisturbed. At t_3_, we flip the initial Δϕ assignment to the electrodes directing the streamline to the upper outlet. A time-lapse movie showing this switching process is provided in Movie S6.

Such flow shaping can also be integrated as part of more elaborated devices. As an example, Fig. 7 shows the use of two electrodes to deflect a central inlet streamline into one of the three possible outlets. At t_1_, the electrodes are set to create a counterclockwise flow, thus deflecting the incoming streamline toward the lower outlet. At t_2_, both the electrodes are set to have a Δϕ=0; the streamline enters the central outlet undisturbed. At t_3_, we set the electrodes to create a clockwise velocity pattern, thus routing the streamline to the upper outlet.

## Conclusion and Outlook

We presented the use of ac-FEEO as a mechanism to create dynamic flow patterns in a Hele–Shaw configuration. We showed that the basic flow pattern of an EOF dipole predicted by the theory can be experimentally reproduced by this mechanism, with dipole strength set by the voltage amplitude difference between the electrode and the bulk. In contrast to zeta-potential modification using chemical patterning (11, 30), electric control allows setting the values of EOF within a continuous range of positive and negative values, and to rapidly switch between them. The timescale for such switching depends on the EDL charging time and the viscous time, which in our system is on the order of 5 ms. We investigated several electrode configurations and demonstrated a variety of flow patterns that can be realized. To the best of our knowledge, some of these patterns, such as the localized recirculation and the stagnation volume, have not been demonstrated by other means. Furthermore, we showed that ac-FEEO can interact with existing flow fields and dynamically tune their streamlines.

Precise microscale flow control may be useful in several applications. For example, whereas large particles can be manipulated in microsystems by other mechanisms such as dielectrophoresis or optical tweezers, controlled transport of small molecules remains challenging. The technique presented here could be particularly useful to bridge this gap, and allow control of the mass transport of small chemicals and biomolecules such as proteins, DNA, peptides, etc. Because this technique drives the fluid itself and not the single particles, it may be useful for heat-transfer management in microdevices, and the pressure field formed may be leveraged to actuate deformable surfaces, such as free surfaces or elastic actuators. In this work, we used few individual electrodes providing access to only a limited set of flow patterns. An ideal flow control system would allow any desired flow pattern on a large scale. One could envision such a system constructed from a large number of individually addressed electrodes, likely in array format.

## Materials and Methods

We characterized the EOF velocity as a function of the applied amplitude difference Δϕ. We used a 100-µm-wide, 15-µm-high, and 1-cm-long straight channel containing an array of gate electrodes distributed over the entire length of the channel and composed of 100 × 100-µm^2^ units, spaced 10 µm edge to edge. These gate electrodes are set to give an equal Δϕ along the channel to ensure a homogeneous EOF slip velocity throughout the channel. We measured the depth-averaged velocity by particle image velocimetry (PIV) analysis, using 0.8-µm carboxyl fluorescent particles (Spherotech Inc.) as flow tracer and PIVlab for the image analysis. Assuming a pure Couette-type flow, we estimate the slip velocity to be twice the measured depth-averaged velocity, and use this to calculate the velocity (shown in Fig. 1*D*) and the EO wall mobility (shown in Fig. 3) via the Helmholtz–Smoluchowski relation.

Additional information on visualization conditions, image analysis, and device fabrication is provided in *SI Appendix*.

## Supplementary Material

Supplementary File

Supplementary File

Supplementary File

Supplementary File

Supplementary File

Supplementary File

Supplementary File
