# Supplementary material for: Dynamic microscale flow patterning using electrical modulation of zeta potential
Source: Proc Natl Acad Sci U S A. 2019 May 6;116(21):10258–63. doi: 10.1073/pnas.1821269116 (PMC6534970; doi:10.1073/pnas.1821269116)
Supplement: Supplementary File [file pnas.1821269116.sapp.pdf]

Supplementary Information for

Dynamic microscale flow patterning using electrical modulation of zeta potential

Federico Paratore[a,b,c], Vesna Bacheva[a], Govind V. Kaigala[a,*], Moran Bercovici[b,c,*]

[a] IBM Research Zurich, Säumerstrasse 4, 8803 Rüschlikon, Switzerland; [b] Faculty of Mechanical Engineering, Technion − Israel Institute of Technology, Haifa, 3200003, Israel; [c] Department of Mechanical Engineering, The University of Texas at Austin, Austin, Texas 78712, USA.

Govind V. Kaigala
Email: gov@zurich.ibm.com

Moran Bercovici
Email: mberco@technion.ac.il

This PDF file includes:

Supplementary text:
1. Experimental visualization and image analysis
2. Device fabrication
3. Time-averaged slip velocity
4. Breakdown characterization

Figs. S1 to S9
Captions for movies S1 to S6
References for SI reference citations

Other supplementary materials for this manuscript include the following:

Movies S1 to S6

www.pnas.org/cgi/doi/10.1073/pnas.1821269116

1. Experimental visualization and image analysis

We used an inverted epi-fluorescence microscope equipped with a solid state light source (Mira, Lumencor), a 10X (NA = 0.45) Nikon PlanApo λ objective, a 4X (NA=0.13) Nikon Plan Fluor objectives, and 0.7X de-magnification lens (Micropix Ltd.) placed in front of the camera. To trace the flow we use 0.8 µm-diameter pink carboxyl fluorescent particles or 80 nm-diamter skyblue carboxyl fluorescent particles (Spherotech Inc.) imaged using an mCherry filter (AHF, 562/40 excitation, 641/75 nm emission, and 593 nm dichroic mirror) and an Cy5 filter (Nikon, 624/40 excitation, 692/40 nm emission, and 660 nm dichroic mirror), respectively. We imaged using a CCD camera (Clara, Andor-Oxford Instrument), using different expsosure times: 1 s for imaging the quadrupoles (Fig. 4, Fig. S7, Movie S2 and Movie S3), 300 ms for imaging the single dipoles (Fig. 1 E, F, Fig. S6 and Movie S1), the flow generated by the two concentric electrodes (Fig. 5, and Movie S4), the shaped streamliens (Fig. 6, Fig. 7, Movie S5 and Movie S6) and for the velocity characterization.

The images showing the experimental streamlines are obtained by superposition of multiple frames after background subtraction. Because the fluorescence intensity in the gate electrode regions is ~ 50 % less than elsewhere, we amplified the signal coming from the electrode regions by a factor of 2 to obtain a uniform signal on the entire image. We then inverted the color of the fluorescence images so that the particles appear in black over a white background. For the velocity measurement (Fig. 1 D and Fig. 3) and for obtaining the vector map in Fig. 1 E and F, we analyze a series of sequential frames using PIVlab (1, 2).

2. Device fabrication

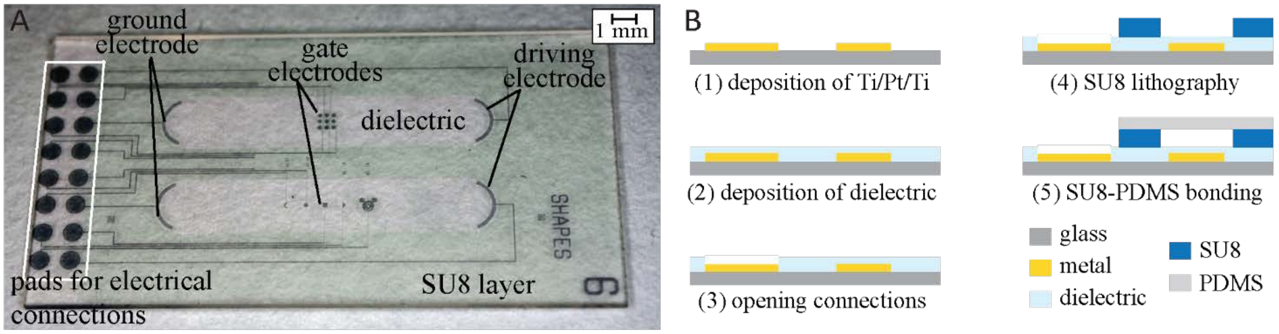

Fig. S1. *Device fabrication. (A) Image of a typical device used in this work comprises two independent microfluidic chambers. We place the gate electrodes at the center of each microfluidic chamber, and connect them through conducting lines to pads at the perimeter of the device, providing an interface to the power supplies. We insulate the gate electrodes by a dielectric layer, leaving the driving, ground and electrical pad electrodes exposed. We use a layer of SU8 to define the lateral walls of the microfluidic chamber, and a ~1 cm thick PDMS slab (not shown in the image) to form the ceiling. (B) Schematic of the microfabrication process. 1. We define the metal structures (2 nm Ti / 2nm Pt / 2 nm Ti) on a glass substrate by a standard lift-off process. 2. We deposit a dielectric layer via PECVD or ALD, and, 3. we open the electrical connections (ground, driving and pads electrode) by wet or dry etching. 4. Using a lithography step we define the microfluidic chambers with a 15 µm thick layer SU8 photoresist. 5. Finally, we form the ceiling of the chambers by using a PDMS slab of approximately 1 cm.*

Fig. S1 A shows a typical device having two independent microfluidic chambers defined by structures in an SU8 layer; each chamber comprises a central region where the gate electrodes (electrically insulated by a dielectric layer) are located, and two open (not covered by the dielectric) electrodes serving as the driving potential and the ground. The electrodes are in contact with external power suppliers through the electrical pads located at one edge of the device.

Fig. S1 B shows the microfabrication process steps. All the microfabrication steps are performed in a cleanroom class 100. We use a 0.5 mm-tick, 4’ wafer double-polished borosilicate glass (Plan Optik AG) as the substrate. We define the metal structures by a lift-off process, depositing a sandwiched metal layer of 2 nm Ti - 2 nm Pt - 2 nm Ti by physical evaporation (BAK501, Evatec AG). We use the two layers of titanium to improve the adhesion of the metal layers to both the substrate and the dielectric; the platinum layer is used because it is

resistant to hydrofluoric acid (HF) and acts as a stopping layer during the etching process for opening the electrical connections. The metal layer thickness is 6 nm, thin enough to allow optical transmittance in the visible spectrum, enabling visualization of fluorescent beads using an inverted epifluorescence microscopy.

We deposit 500 nm of silicon oxynitride (SiON) followed by 100 nm of silicon dioxide (SiO_2) as dielectric layer by plasma-enhanced chemical vapor deposition (PECVD). To test different dielectric materials (see section ‘System design and characterization’ in the main text and Fig. S8 for more details) we use either PECVD, for SiO_2, SiON and silicon nitride (SiN_x), or atomic layer deposition (ADL), for SiO_2, hafnium dioxide (HfO_2) and aluminum oxide (Al_2O_3). We then expose the electrical contact by etching the dielectric over the driving electrodes and the pads by using HF (for SiO_2, SiON, HfO_2 and Al_2O_3) or dry etching for SiN_x.

We deposit a 15 µm-thick layer of SU8 (SU8 3010, MicroChem Corp.) by spinning it for 40 s at 1500 rpm, and define the lateral walls of the micofluidic structure by a standard lithoghrapic step. Importantly, during all thermal steps (soft- and post-baking) for the SU8 treatment we do not elevate the temperature above 95 ˚C to keep residual epoxy groups active (3). We dice the wafer to singulate the devices with dimensions of approximately 2 × 1 cm.

We produce PDMS (Sylgard 184, Dow Corning) slabs of approximately 1 cm using a cross-linker to monomer ratio of 1:10 and cure it at 60°C for 3 h. We create the reservoirs by punching through the PDMS using standard circular biopsy punches (1 to 4 mm diamater). Finally, we treat the PDMS with air plamsa at 100 W for 30 s, place it in contact with the SU8 and bake at 140 ˚C to ensure a permanent bonding.

Both the driving and gate potentials were generated by one ore more high-voltage power supplies (2410, Keithley) triggered in sync by a waveform generator. The gate potential is tuned dynamically by using a voltage divider, as shown in Fig. S9 in the SI. Unless specified otherwise, we used an electrolytecomposed of 10 mM acetic acid and 1 mM NaOH (pH 3.8). In the streamline shaping experiments, we generated pressure driven flow by applying negative pressure to one of the reservoirs using a water column.

3. Time-averaged slip velocity

Let us consider the case of a uniform surface, having an embedded electrode (hereafter referred to as 'gate electrode'), covered with an electrolyte and subject to a periodic electric field $E(t)=V_{ex}(t)/L$, where $V_{ex}(t)=\phi_{ex}f(\omega_{ex}t)+C_{ex}$ is the potential on the driving electrode, placed at a distance L from the ground electrode. Here, ϕ_{ex} is the amplitude of the potential, $f_{ex}=1/T_{ex}$ is the frequency and T_{ex} is the period. The gate electrode, placed at a distance x_{el} from the ground electrode, is subject to a periodic potential $V_{el}(t)=\phi_{el}f(\omega_{el}t+\Omega)+C_{el}$, where ϕ_e is the amplitude of the potential, $\omega_{el}=1/T_{el}$ is the frequency and T_{el} is the period, Ω is the phase difference between $V_{el}(t)$ and $V_{ex}(t)$, and C_{el} is a DC bias potential. We assume the electrode is small compared to the length of the channel L, and thus the potential in the liquid above the electrode is $V_{ch}(t)=(x_{el}/L)V_{ex}(t)$. The electroosmotic flow velocity generated by the surface over the electrode is then given by the Helmholtz–Smoluchowski slip velocity

$$u_{EOF}=-\frac{\varepsilon\zeta(t)}{\eta}E(t) \quad \text{[S1]}$$

where ε_l and η are the dielectric permittivity and viscosity of the liquid respectively, and $\zeta(t)$ is the time variant zeta potential which is some function of the potential drop across the dielectric $\zeta(t)=G(V_{el}(t)-V_{ch}(t))$. Substituting the value for $E(t)$ and $\zeta(t)$ in Eq. S1 we obtain

$$u_{EOF}(t)=-\frac{\varepsilon_l}{\eta L}\left(\phi_{ex}f(\omega_{ex}t)+C_{ex}\right)G\left(\phi_{el}f(\omega_{el}t+\Omega)+C_{el}-\frac{x_{el}}{L}\phi_{ex}f(\omega_{ex}t)-\frac{x_{el}}{L}C_{ex}\right), \quad \text{[S2]}$$

which when integrated over the lowest common multiple period T^{lcm} yields the time averaged velocity

$$u_{EOF}^{av}=\int_0^{T^{lcm}}u_{EOF}(t)dt\ . \quad \text{[S3]}$$

Here, we consider the simplified case where the electric fields and the zeta potentials have the same period $\omega=\omega_{el}=\omega_{ex}$ (assumed to be smaller than the inverse RC time scale of the electrical system $\tau_{RC}{}^{-1}$), and zero phase difference $\Omega=0$, resulting in a maximal time-averaged EOF velocity (4). Further, we consider the case where $f(\omega t)$ is a square wave (i.e. $f(\omega t)=1$ and $f(\omega t)=-1$ for the first and second half of the period, respectively). Defining the difference between the gate potential and the channel potential amplitudes as

$\Delta\phi = \phi_{el} - (x_{el} / L)\phi_{ex}$, and the difference between the DC bias on the gate potential and in the channel as $\Delta C = C_{el} - (x_{el} / L)C_{ex}$, the EOF velocity (Eq. S3) becomes

$$u_{EOF}(t) = \begin{cases} u_{EOF}^{T/2} = -\dfrac{\varepsilon_l}{\eta L}\phi_{ex} G\left(\Delta\phi + \Delta C\right) - \dfrac{\varepsilon_l}{\eta L} C_{ex} G\left(\Delta\phi + \Delta C\right) \\ u_{EOF}^{T} = \dfrac{\varepsilon_l}{\eta L}\phi_{ex} G\left(-\Delta\phi + \Delta C\right) - \dfrac{\varepsilon_l}{\eta L} C_{ex} G\left(-\Delta\phi + \Delta C\right) \end{cases}, \qquad \text{[S4]}$$

where the superscripts $T/2$ and T indicate the first and second half of the period, respectively. Thus the time averaged velocity (Eq. S4) is simply the arithmetic mean of the velocity of the two half periods

$$u_{EOF}^{av} = \underbrace{-\frac{1}{2}\frac{\varepsilon_l}{\eta L}\phi_{ex}\left(G(\Delta\phi + \Delta C) - G(-\Delta\phi + \Delta C)\right)}_{\text{AC contribution}} \underbrace{-\frac{\varepsilon_l}{\eta L} C_{ex} \frac{G(\Delta\phi + \Delta C) + G(-\Delta\phi + \Delta C)}{2}}_{\text{DC contribution}}. \qquad \text{[S5]}$$

The time averaged velocity is composed of two terms corresponding to an AC contribution which depends on the difference of the zeta potential values between the two half periods, and a DC component, which depends on their sum. When no DC bias is applied to either the gate electrode or driving electrode, i.e. $C_{ex} = C_{el} = 0$, then the averaged slip velocity depends only on the amplitude differences $\Delta\phi$ and the magnitude of the driving field ϕ_{ex},

$$u_{EOF}^{av}(\Delta\phi) = -\frac{1}{2}\frac{\varepsilon_l}{\eta L}\phi_{ex}\left[G\left(\Delta\phi\right) - G\left(-\Delta\phi\right)\right]. \qquad \text{[S6]}$$

4. Breakdown characterization

We characterized the dielectric breakdown of various dielectric materials and layer thickness using the configuration depicted in Fig. S2, in which an electrode immersed in a solution of 10 mM lactic acid and 5 mM NaOH (pH 3.7) is in contact with a grounded gate electrode (covered with the specific dielectric under study).

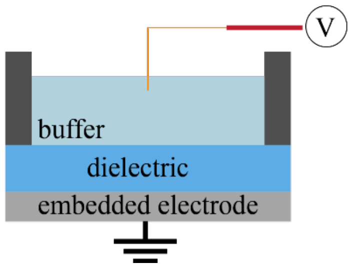

Fig. S2. Schematic *of the experimental setup for the breakdown experiments. The dielectric deposited on top of a gate electrode is immersed in a buffer composed of 10 mM lactic acid and 5 mM NaOH (pH 3.7). We apply an electric field across the dielectric and monitor the current. The electric field is increased, until the dielectric breaks.*

We apply an increasing ($\Delta\phi < 0$)[1] or decreasing ($\Delta\phi > 0$) potential with steps between 10 and 50 V every 3 s, while monitoring the I-V curve using a high voltage power supply (Keithley, 2410) controlled with Matlab (R2017, MathWorks). We observed two typical behaviors of the I-V curve. The first type, shown in Fig. S3 A, is characterized by a drastic increase of the current beyond a certain threshold, which we attribute to the dielectric breakdown. This type of behavior is common for SiO_2 and therefore we define this response SiO_2-type. For this type, we define the breakdown value as the first voltage at which two subsequent measurements yield a current increase of more than 10-fold. The other type, shown in Fig. S3 B, is characterized by a smooth I-V We found this type of curves to be typical of SiN-based dielectrics, and thus we refer to them as SiN-type.

---

[1] $\Delta\phi$ is defined as the gate potential minus the potential in the channel (i.e. $\Delta\phi = \phi_{el} - \phi_{ch}$). Because in this configuration the electrode is grounded, increasing applied potentials correspond to negative $\Delta\phi$.

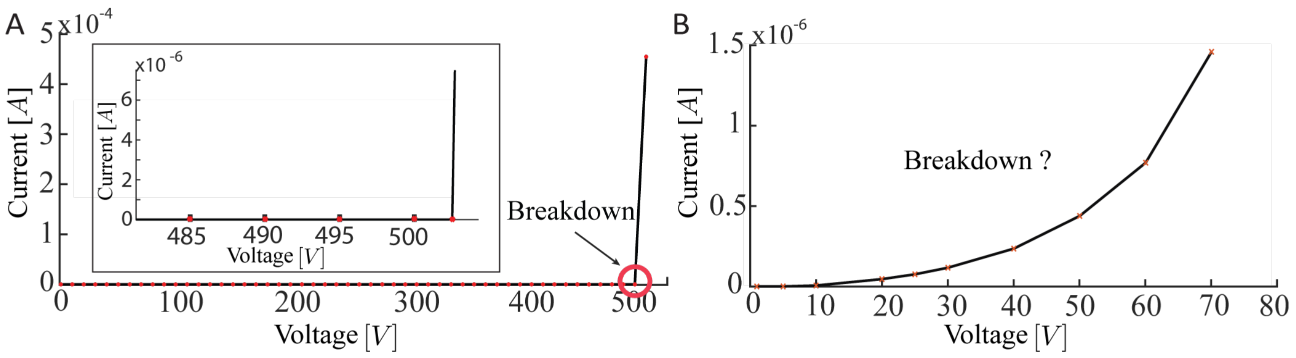

Fig. S3. *I-V measurements of the dielectric. (A) Current profile of SiO2 in function of the voltage applied across the dielectric. The current is zero until the breakdown when it increases drastically. (B) Current profile of SiN. The current constantly increases, without abrupt change.*

Whereas for the SiO_2-type the value of the breakdown can be directly extract from the observation of the I-V curve, for the SiN-type we assessed the breakdown values performing a series of hysteresis cycles: for each voltage value being tested, we ramp the voltage in 1 V steps until reaching the tested voltage, and then ramp down the voltage at the same rate. We define the breakdown voltage as the lowest test voltage for which the ramp down curve no longer follows the ramp up curve. Fig S4 A and B show the two curves for a test voltage below (20 V) and after (40 V) breakdown, respectively.

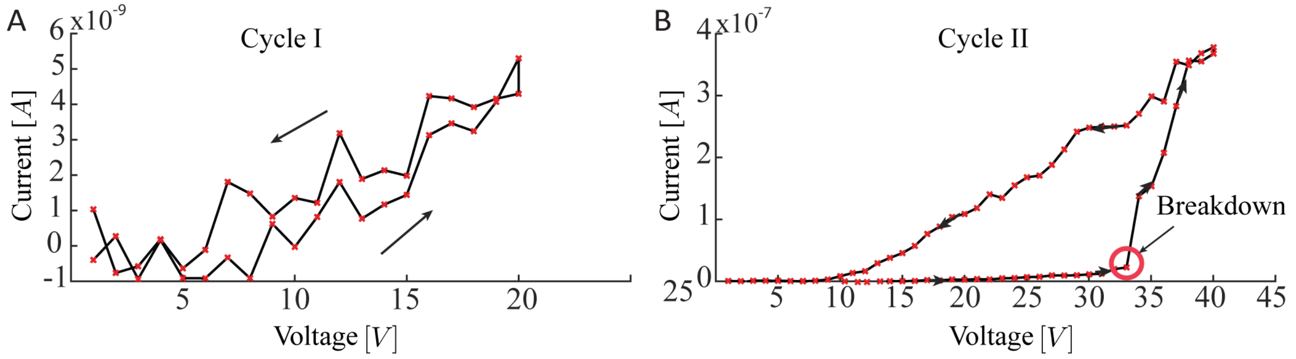

Fig. S4. *Typical I-V hysteresis measurements for a dielectric showing SiN-type behavior using a 1 V scanning step. (A) The voltage was increased up to 20 V, and then decreased with the same scanning rate. The current shows a similar trend for increasing and decreasing voltages, indicating that the breakdown voltage has not been reached. (B) The same material is subject to second cycle of hysteresis where the voltage was increased up to 40 V, showing a significant change in the I-V curve between the up and down voltage ramping, indicating a change of the electric properties of the dielectric, thus the breakdown of the material.*

After performing such hysteresis experiments on three SiN–coated devices, we concluded that the average value of the current at which the breakdown occurs is ~ 10^{-7} A and we used this value as a decision point for all SiN-type dielectrics. The dielectric coatings tested in this work showing SiO_2-type are HfO_3 + SiO_2, SiO_2+SiO_2, Al2O_3+SiO_2 for the family of layers consisting of 20 nm deposited with ALD followed by 500 nm deposited with PECVD (Fig. S8) and SiO_2, SiN (500nn) + SiO_2 (500nm), SiN_x (200nm) + SiO_2 (300nm), SiO_2 (200 nm)+SiN_x (300 nm), SiON (500 nm), SiO_2 TEOS (500 nm), SiO_2 TEOS (200 nm) + SION (300 nm) for the family of layers deposited with PECVD (Fig. 2 A in the main text). The dielectric coatings tested in this work showing SiN-type are HfO_3 + SiN_x, Al_2O_3 + SiN_x, SiO_2 (ALD) + SiN_x for the family of layers consisting of 20 nm deposited with ALD followed by 500 nm deposited with PECVD (Fig. S8) and SiN (500nn) deposited with PECVD (Fig. 2 A in the main text).

5. Supplementary figures

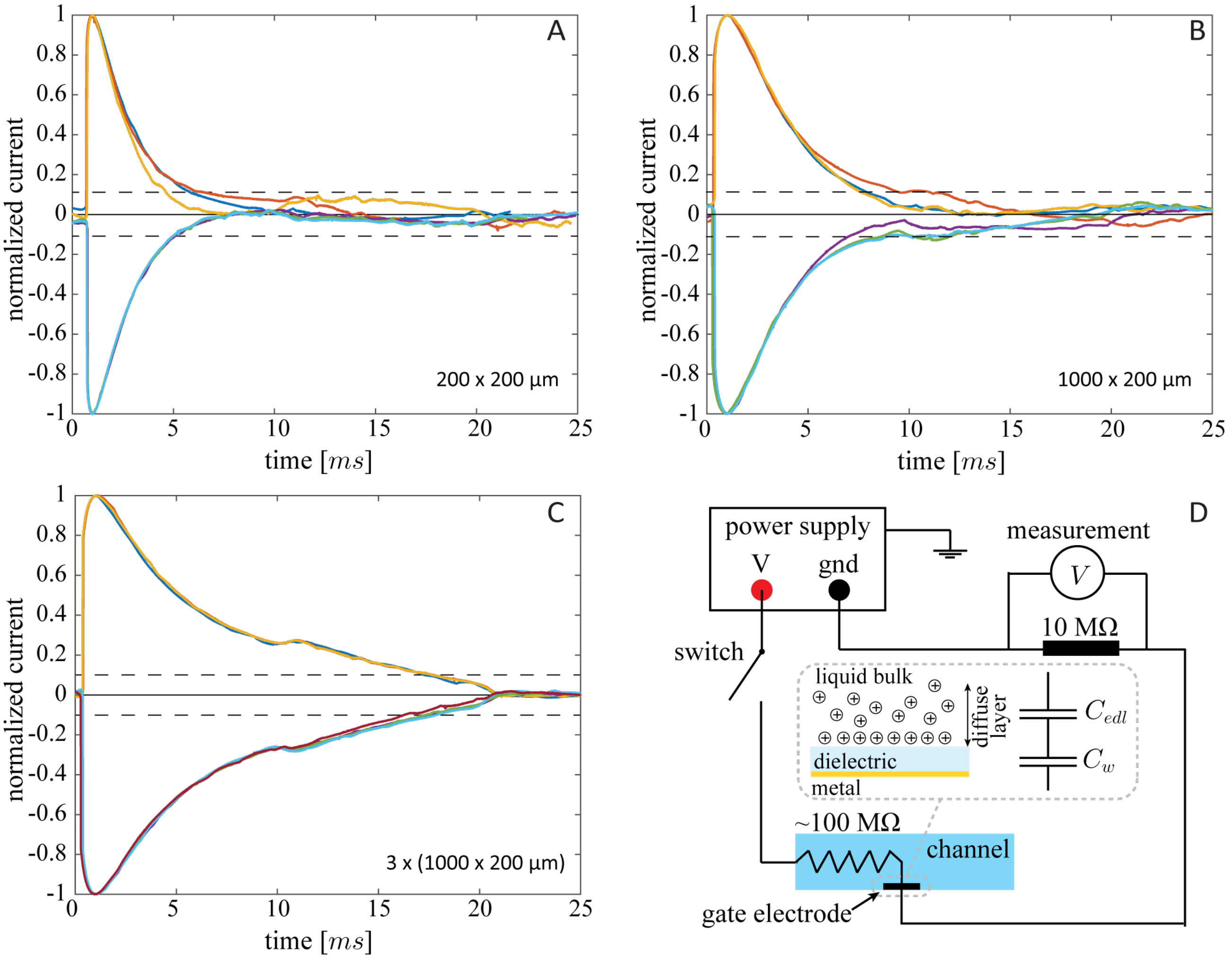

Fig. S5. *We characterized the dynamic response of our system by imposing a step function in potential and measuring the potential drop over an external reference resistor in series with our circuit, indicating the current flowing through the electrode. (D) Our system comprises a microfluidic channel filled with a buffer solution composed of 10 mM of acetic acid and 1 mM NaOH 1mM (pH 3.8), one or more gate electrodes, a reference resistor of 10 MΩ and a measuring instrument. The channel acts as an equivalent resistor of approximately 100 MΩ (as measured in separate experiments), and the gate electrodes as an equivalent capacitor, thus forming an equivalent RC system. To monitor and analyze the potential across the reference resistor we use a Multifunction I/O Device (National Instrument, UBS 6211) controlled with LabView (National Instruments). To impose the sudden change in potential, we set the desired DC voltage (100V or -100V) on the power supply*

and use an external mechanical switch to close the system, thus eliminating any RC effect associated with the power supply internal circuitry. Defining the RC time constant as the time point where the voltage over the reference resistor drops by 90% of its initial value, we characterized the RC response for gate electrodes with sizes of 200 x 200 µm (A), 1000 x 200 µm (B) and 1000 x 200 µm (C), obtaining values of the time constant of 5 ms, 8.5 ms and 17 ms, respectively.

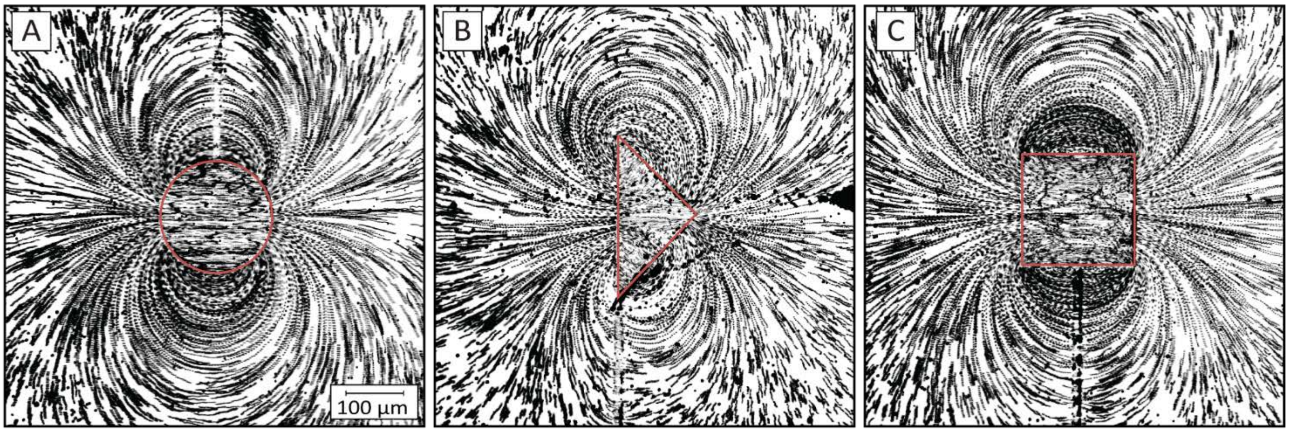

Fig. S6 *Experimental visualization of flow streamlines for different isolated gate electrode geometries. The external amplitude is* $\phi_{ex} = 200V$ *, resulting in a voltage in the bulk on the electrodes of approximately* $\phi_{ch} = 100V$ *. A disk-shaped electrode (A) gives rise to an electroosmotic dipole, well predicted by the theory (Eq. 4 in the main text)* (5)*; see Movie S1 for a continuous experimental visualization. Other isolated gate electrodes with different shapes, such as a triangle (B) and a square (C), exhibit a similar behavior, characterized by recirculating flow at the top and bottom with zero net mass flux.*

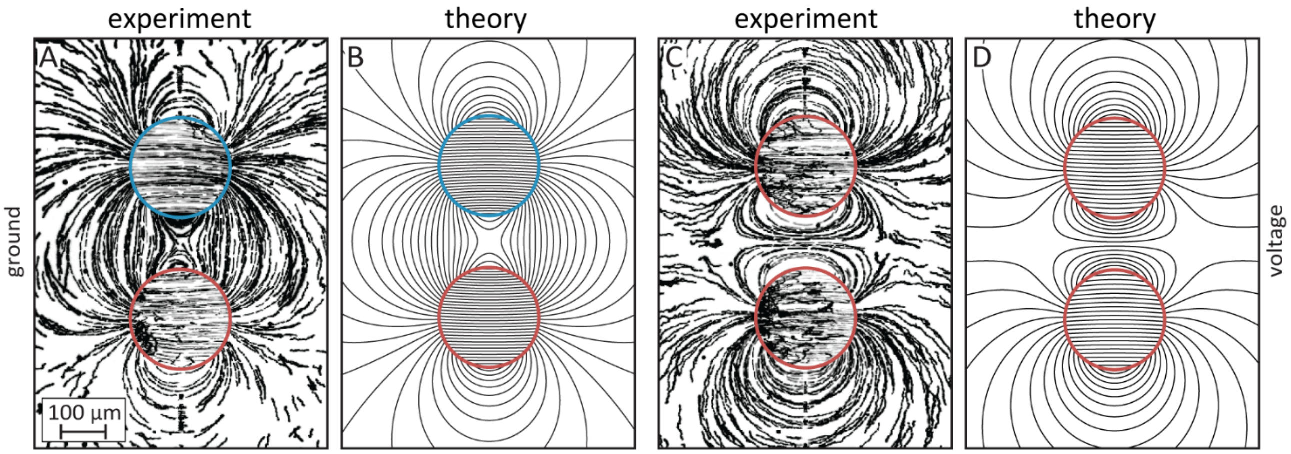

Figure S7. *Analytical predictions and experimental visualization of flow streamlines generated by two 200 µm-diameter disk-shaped gate electrodes for different* $\Delta\phi$ *combinations. The external amplitude is* $\phi_{ex} = 200V$ *, resulting in a bulk voltage over the electrodes of approximately* $\phi_{ch} = 100V$ *. (A, B) We set the amplitude difference of the bottom and top electrode to* $\Delta\phi = -80\,V$ *and* $\Delta\phi = 80\,V$ *, respectively, giving rise to a counter clockwise flow field. (C, D) At a later moment, we switch the potential of the top electrode to match the bottom one* $\Delta\phi = 80\,V$ *, resulting in a flow field similar to an electroosmotic flow dipole in the far field with internal recirculation between the two disks. See Movie S3 for the corresponding video.*

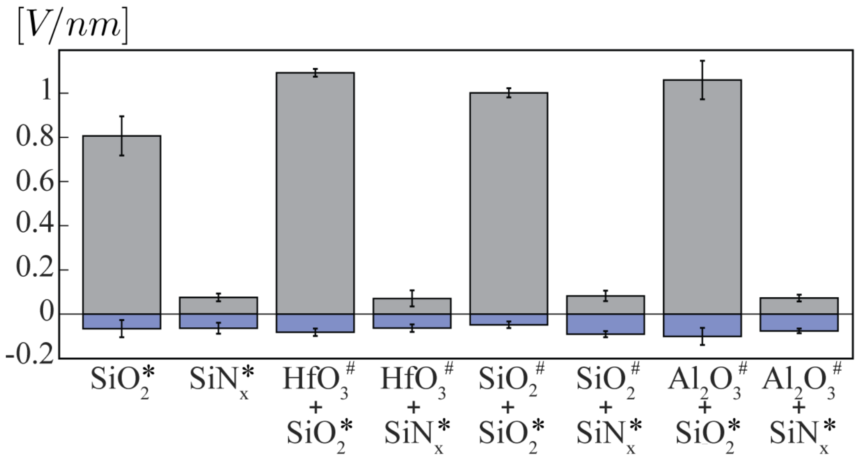

Fig. S8. *Experimental characterization of the breakdown strength of 500 nm dielectric layers of SiO_2 or SiN deposited with PECVD (indicated with an asterisk) on top of 20 nm layer of different material deposited with ALD layer (indicated with a hash). At positive applied potentials ($\Delta\phi > 0$), pure SiO_2 performs significantly better than SiN_x, showing a breakdown strength of ~0.8 V/nm and ~0.1 V/nm, respectively. At negative applied potentials ($\Delta\phi < 0$), they show a poor breakdown strength of ~0.1 V/nm. This clear asymmetry of the SiO_2 response is enhanced by the use of a layer of 20 nm deposited with ADL, which boosts the strength of ~20% for positive potentials, but is unable to improve the performance for negative voltage. Adding this additional layer of 20 nm does not improve the performance of SiN_x coatings. The error bars represent the 95% confidence interval of the mean (with n at least 10).*

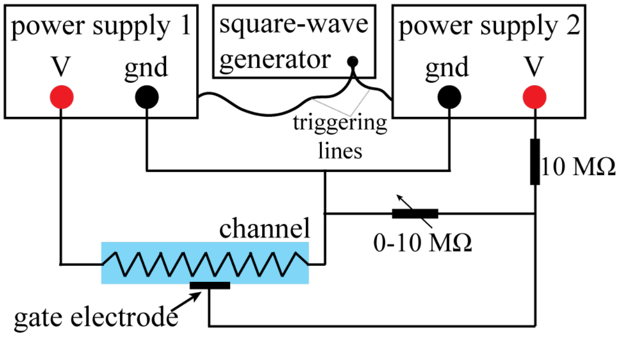

Fig S9. *Illustration of the electric circuit we use to drive the field in the channel and set the gate potential. Power supply 1 is directly connected to the two reservoirs of the chamber, and drives the AC field through the channel. The voltage output of power supply 2 is connected to the ground through a fixed 10 MΩ resistor in series with a variable one, 0 to 10 MΩ. The gate electrode (which may be considered as an equivalent capacitor) is placed in parallel with the variable resistor. In this way, tuning the resistance of the variable resistor we are able to modify dynamically the gate potential, and thus the EOF velocity.*

6. Caption for movies

Movie S1. Variable Electrosomotic Flow Dipole. *A single disk-shaped electrode gives rise to an electroosmotic flow dipole flow. We control the flow magnitude and direction by modulating the potential of the electrode. Each frame of the video was background subtracted and obtained by the superposition of 10 frames of the raw data time-lapse.*

Movie S2. Variable Electroosmotic Flow Quadrupole: horizontal configuration. *Fluorescence raw data time lapse showing the motion of 0.8 µm-diameter fluorescent in flow patterns resulting from two disk-shaped electrodes placed along the electric field. By modulating the potentials of the electrodes, a range of flow patterns can be realized, including classical quadrupole field and nested dipole configuration.*

Movie S3. Variable Electroosmotic Flow Quadrupole: vertical configuration. *Fluorescence raw data time laps showing the motion of 0.8 µm-diameter fluorescent beads in flow patterns resulting from two disk-shaped electrodes placed perpendicular to the electric field. By modulating the potentials of the electrodes, a range of flow patterns can be realized.*

Movie S4. Variable Flow Field using Concentric Electrodes. *A disk-shaped electrode surrounded by an annulus shaped electrode can be configured to yield unique flow fields: recirculating flow within a finite region surrounded by quiescent fluid, and a finite stagnation volume within a flow field. The video shows the fluorescent raw data time lapse of the motion of 0.8 µm-diameter fluorescent beads under such flow fields.*

Movie S5. Streamline Shaping. *Flow shaping using an array of electrodes. An incoming streamline generated by pressure driven flow is dynamically deformed by actuation of the electrodes. The streamline is visualized by using 80 nm-diameter fluorescent beads as tracer.*

Movie S6. Flow Switching by Streamline Shaping. *A central incoming streamline is directed towards one of the three outlets by controlling two gate electrodes disposed perpendicularly to the electric field. The streamline is visualized by using 80 nm-diameter fluorescent beads as tracer.*

7. References

1. Thielicke W, Stamhuis E (2014) PIVlab – Towards User-friendly, Affordable and Accurate Digital Particle Image Velocimetry in MATLAB. *Journal of Open Research Software* 2(1):e30.

2. Thielicke W, Stamhuis EJ (2018) PIVlab - Time-Resolved Digital Particle Image Velocimetry Tool for MATLAB (version: 1.41). doi:10.6084/m9.figshare.1092508.v10.

3. Zhang Z, Zhao P, Xiao G, Watts BR, Xu C (2011) Sealing SU-8 microfluidic channels using PDMS. *Biomicrofluidics* 5(4):046503-046503–8.

4. Kuo C-Y, Wang C-Y, Chang C-C (2008) Generation of directional EOF by interactive oscillatory zeta potential. *ELECTROPHORESIS* 29(21):4386–4390.

5. Boyko E, Rubin S, Gat AD, Bercovici M (2015) Flow patterning in Hele-Shaw configurations using non-uniform electro-osmotic slip. *Phys Fluid* 27(10):102001.
